# A ^13^C-detected ^15^N double-quantum NMR experiment to probe arginine side-chain guanidinium ^15^N^η^ chemical shifts

**DOI:** 10.1007/s10858-017-0137-2

**Published:** 2017-11-10

**Authors:** Harold W. Mackenzie, D. Flemming Hansen

**Affiliations:** Institute of Structural and Molecular Biology, Division of Biosciences, University College London, London, WC1E 6BT UK

**Keywords:** 13-Carbon-detected NMR, Arginine side-chains, Double-quantum coherence, Conformational exchange, Isotope shifts

## Abstract

Arginine side-chains are often key for enzyme catalysis, protein–ligand and protein–protein interactions. The importance of arginine stems from the ability of the terminal guanidinium group to form many key interactions, such as hydrogen bonds and salt bridges, as well as its perpetual positive charge. We present here an arginine ^13^C^ζ^-detected NMR experiment in which a double-quantum coherence involving the two ^15^N^η^ nuclei is evolved during the indirect chemical shift evolution period. As the precession frequency of the double-quantum coherence is insensitive to exchange of the two ^15^N^η^; this new approach is shown to eliminate the previously deleterious line broadenings of ^15^N^η^ resonances caused by the partially restricted rotation about the C^ζ^–N^ε^ bond. Consequently, sharp and well-resolved ^15^N^η^ resonances can be observed. The utility of the presented method is demonstrated on the L99A mutant of the 19 kDa protein T4 lysozyme, where the measurement of small chemical shift perturbations, such as one-bond deuterium isotope shifts, of the arginine amine ^15^N^η^ nuclei becomes possible using the double-quantum experiment.

**Electronic supplementary material:**

The online version of this article (doi:10.1007/s10858-017-0137-2) contains supplementary material, which is available to authorized users.

## Introduction

Of the twenty standard proteinogenic amino acids that are found in nature few are as important as arginine. This essential amino acid is found at many protein interaction surfaces (Crowley and Golovin 2005; Rohs et al. 2010) and has been identified in numerous enzymatic active sites (Casey et al. 2014; Friedt et al. 2014; Zeymer et al. 2016) and substrate-binding pockets (Goldschen-Ohm et al. 2011; Gargaro et al. 1996). The importance of the arginine side-chain for protein functions stems from its terminal guanidinium group that is the most basic moiety of the three positively charged amino acids. The high pK_a_ (~ 14) (Fitch et al. 2015) of the guanidinium group renders the arginine side-chain positively charged at all physiologically relevant pHs (Harms et al. 2011) and thereby provides nature with a reliable means of placing a positive charge at virtually any point within a protein structure. An important feature of the arginine guanidinium group is that the positive charge is delocalised and the arginine side-chain is therefore capable of an impressive range of hydrogen bonds and ionic interactions. These interactions include bidentate salt bridges with carboxylates and phosphates, cation–π interactions with aromatic rings and hydrogen bonding with the side-chain groups of aspartic and glutamic acids as well as with backbone carbonyl oxygens (Borders et al. 2008; Nieto et al. 1997).

Solution state nuclear magnetic resonance (NMR) spectroscopy is well positioned to characterise the interactions formed by arginine side-chains due to the atomic resolution the technique can provide. Protein side-chains and their interactions are inherently dynamic and whilst NMR spectroscopy is considered suitable for the study of such systems, it is often the case that dynamic processes obscure the underlying information (Kleckner and Foster 2011). For example, the NMR signals of interest are often severely broadened when states are interconverting with rates that are comparable to the difference in chemical shift between the exchanging states. A specific example is encountered for the terminal –N^η^H_2_ amines of the arginine side-chain (Yamazaki et al. 1995; Yoshimura et al. 2017; Henry and Sykes 1995). The partial double-bond character of the C^ζ^–N^ε^ bond in the guanidinium group causes a decrease in the rotational frequency about this bond, which leads to significant exchange-broadening of the NMR signals associated with the ^15^N^η^ nuclei (Nieto et al. 1997). Moreover, the –N^η^H_2_ amine protons often exchange rapidly with the bulk solvent at physiological pH (Henry and Sykes 1995). Thus, the interconversion of the two ^15^N^η^ nuclei combined with the exchange of the amino protons with the bulk solvent often result in ^1^H^η^–^15^N^η^ NMR correlations that are so broad that very limited information can be gleaned.

The line broadening induced by chemical exchange is reduced when the difference in chemical shift between the exchanging sites, Δω, is reduced (McConnell 1958; Hansen and Led 2003). Many NMR experiments have been developed over the last few decades to manipulate the effective line broadening of chemically exchanging nuclei in a quantitative manner by reducing the effective chemical shift difference (Palmer 2014; Korzhnev et al. 2008; Farber and Mittermaier 2015; Hansen et al. 2008; Zhuravleva et al. 2008; Carr and Purcell 1954; Palmer and Massi 2006). Also, different line broadenings are generally observed for zero-, single-, double- and triple-quantum coherences of a spin system, because these coherences have different precession frequencies and therefore different Δω (Yuwen et al. 2016; Orekhov et al. 2004; Pervushin et al. 1999). A major motivation of the presented work is to characterise the interactions formed by arginine side-chains by generally allowing for an observation of the ^15^N^η^ chemical shifts. Rather than quantifying the chemical exchange, as has been done previously (Henry and Sykes 1995; Gerecht et al. 2017), our focus here is to eliminate the effects of chemical exchange in order to obtain NMR correlation spectra of arginine ^15^N^η^ nuclei.

Below we describe an experiment based on ^13^C detection and the evolution of a double-quantum ^15^N^η^ coherence that overcomes the hurdles associated with the line broadening caused by the rotation about the C^ζ^–N^ε^ bond and the exchange of the ^1^H^η^ protons with the bulk solvent. We also present an application to the 19 kDa protein T4 lysozyme, where the double-quantum experiment allowed the observation of ^13^C^ζ^–^15^N^η^(DQ) coherences for all 13 arginine side-chains. The increased resolution provided by this experiment allows the measurement of small chemical shift perturbations of the arginine terminal amines, demonstrated here with a determination of the deuterium isotope shifts (Hansen 2000) which are expected to inform on salt-bridges and hydrogen bonding in biomolecules (Tomlinson et al. 2009; Williamson et al. 2013).

## Materials and methods

### Protein preparation

Uniformly labelled [^13^C, ^15^N]-T4 Lysozyme L99A was overexpressed and purified from *Escherichia coli*. BL21 (DE3) cells were grown at 37 °C in M9 minimal media supplemented with 1 g/L ^15^NH_4_Cl and 3 g/L [^13^C_6_]-glucose as the sole nitrogen and carbon sources. The expression and purification was performed as described previously (Vallurupalli et al. 2009) with minor modifications. Cells were induced with 1 mM IPTG at OD_600_ of ~ 1.0 before protein expression was allowed to proceed at 16 °C. Cells were harvested by centrifugation after 16 h. The resulting cell pellet was re-suspended and lysed by sonication before purification by ion-exchange (IEX) and gel-filtration (GF) chromatography. ESI-MS confirmed the sample was isotopically enriched to a level beyond 99.7%. The sample was exchanged into NMR buffer (50 mM sodium phosphate, 25 mM NaCl, 2 mM EDTA, 2 mM NaN_3_, pH 5.5, 1% D_2_O) and concentrated to ~ 2 mM. Samples for the measurement of deuterium isotope shifts were prepared by spiking the above samples with additional D_2_O to a final concentration of 10 vol.%.

### NMR spectroscopy

**Fig. 1:**
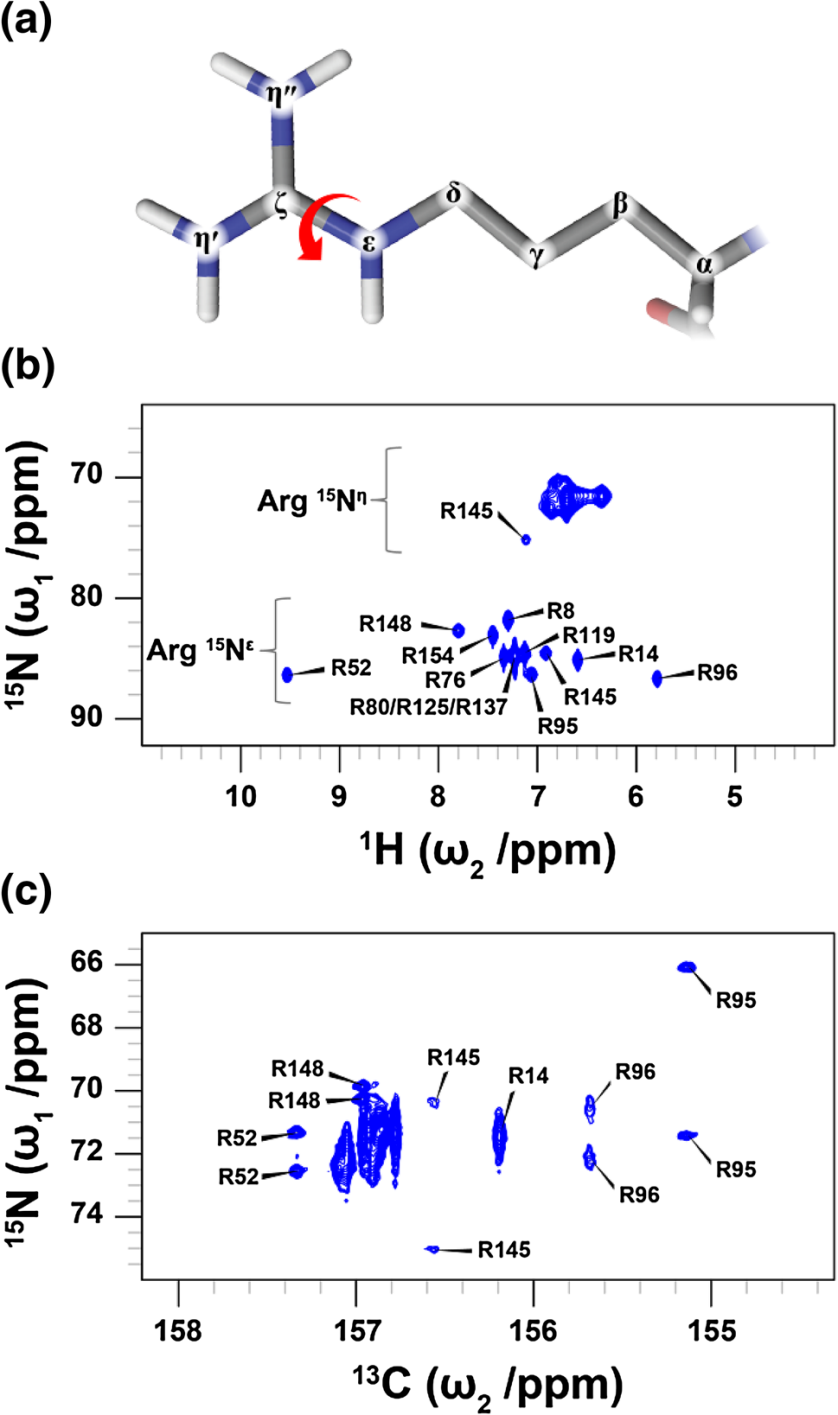
**a** Chemical structure of the arginine side-chain. The arrow highlights the C^ζ^–N^ε^ bond, about which rotation is partially restricted. **b** ^1^H–^15^N HSQC spectrum with focus on the arginine side-chains of T4L99A recorded at 11.7 T. Whilst the majority of the ^15^N^ε^ resonances are detectable, the peaks associated with the amine ^15^N^η^ are broad and overlap significantly. **c** ^13^C^ζ^–^15^N^η^ HSQC spectrum of T4L99A recorded at 16.4 T. Arginine residues that exhibit slow rotation about the C^ζ^–N^ε^ bond give rise to two sharp peaks (R52, R95, R145 and R148). For many of the arginine side-chains, the rotational frequency about the C^ζ^–N^ε^ bond approaches the chemical shift difference between the two ^15^N^η^ causing a substantial line broadening of the NMR signals

**Fig. 2:**
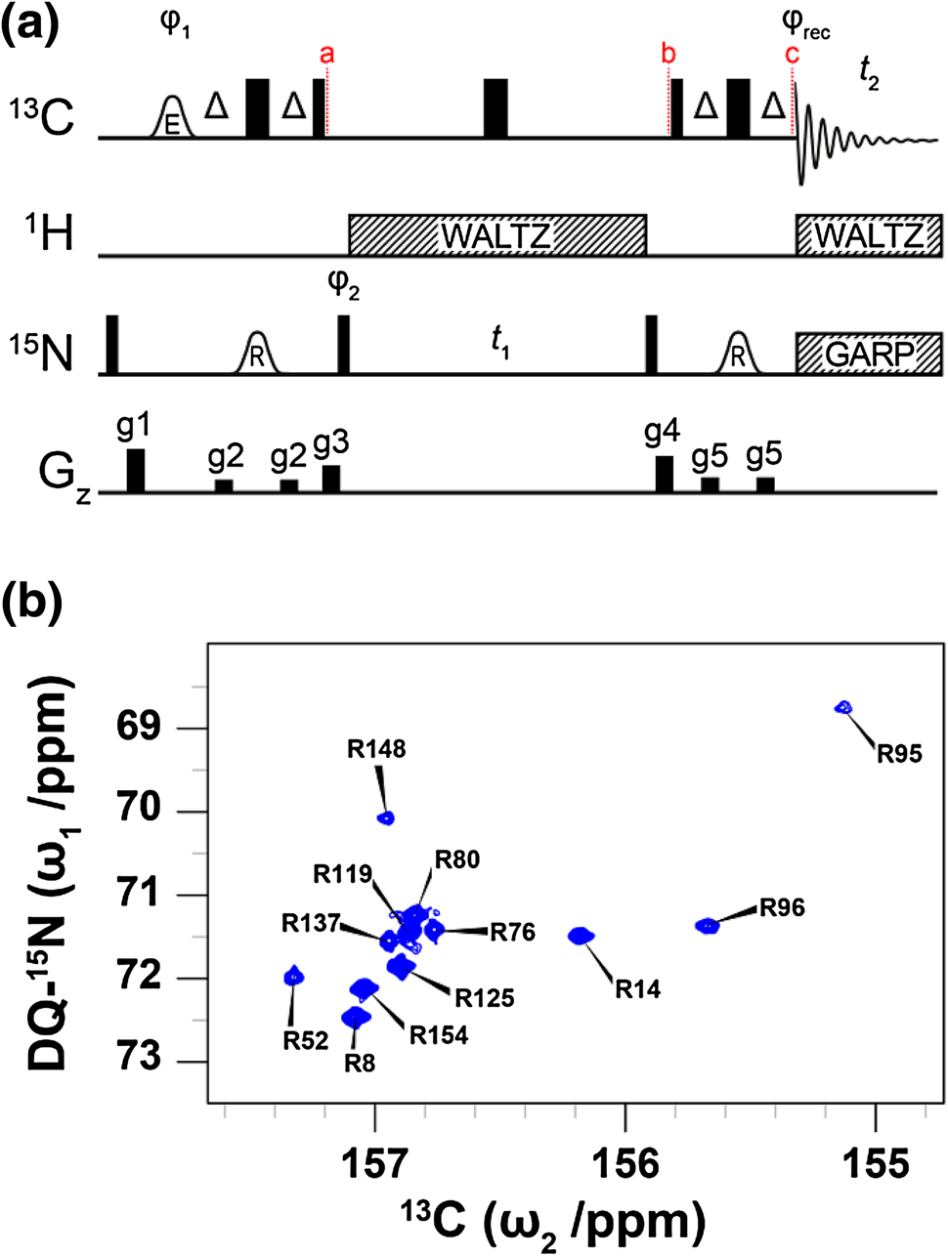
**a** Pulse sequence for obtaining ^13^C^ζ^–^15^N^η^ double-quantum correlation (HDQC) spectra of arginine side-chains in proteins. The carrier positions are ^13^C: 156 ppm, ^15^N: 71 ppm (78 ppm decoupling) and ^1^H: 7 ppm. Narrow and wide bars represent 90° and 180° pulses, respectively, and are applied at maximum power. The delay Δ is 1/(4*J*_CN_) = 12.5 ms. Shaped pulses are represented by bell shapes with letters indicating the shape of the pulse [E: E-BURP-2, R: RE-BURP (Geen and Freeman 1991)] and are applied with durations of 1.5 ms (E) and 4.5 ms (R) at 16.4 T. Pulses are applied with *x* phase unless stated otherwise. The phase cycle used is φ_1_: *x, − x*, φ_2_: 2(*x*), 2(*y*), 2(*− x*), 2(*− y*), φ_rec_: *x*, 2(*− x*), *x*. Decoupling sequences are represented by striped boxes indicating the type of decoupling: WALTZ64 (Shaka et al. 1983; Zhou et al. 2007) (4 kHz), GARP4 (Shaka et al. 1985) (0.7 kHz). Gradient pulses of 1 ms are represented by black rectangles and applied with strengths of g1: 19.8 G/cm, g2: 5.9 G/cm, g3: 12.3 G/cm, g4: 16.6 G/cm, g5: 7.0 G/cm. **b** ^13^C^ζ^–^15^N^η^ HDQC spectrum of T4L99A recorded at 16.4 T. The indirect ^15^N chemical shift is encoded by the double-quantum components $4C_{z}^{\zeta }N_{+}^{{{{{\eta}1}}}}N_{+}^{{{{{\eta}2}}}}$ and $4C_{z}^{\zeta }N_{ - }^{{{{{\eta}1}}}}N_{ - }^{{{{{\eta}2}}}}$ and processed such that the cross peaks appear at the average chemical shift of the two contributing ^15^N^η^ frequencies

All NMR experiments were carried out at 25 °C on Bruker Avance III(HD) spectrometers with ^1^H operating frequencies of 500, 700 and 800 MHz and equipped with helium-cooled TCI (700, 800 MHz) or nitrogen-cooled Prodigy (500 MHz) inverse cryoprobes. The ^1^H–^15^N HSQC spectrum (Fig. 1b) was acquired as a 1024 × 512 complex matrix with spectral widths of 16 ppm (^1^H) and 80 ppm (^15^N). Adiabatic ^13^C decoupling was applied during *t*_1_ and suppression of the H_2_O resonance was achieved using a water-selective flip-back pulse (Andersson et al. 1998) immediately after the first INEPT element. Four scans were collected for each *t*_1_ increment with a recycle delay of 1 s resulting in a total experiment time of 1 h 20 min. Both the ^13^C^ζ^–^15^N^η^ HSQC (Fig. 1c) and HDQC (Fig. 2b) spectra were acquired as 512 × 48 complex matrices with spectral widths of 11 ppm (^13^C) and 12 ppm (^15^N). 256 scans were collected for each *t*_1_ increment with a recycle delay of 3 s resulting in a total acquisition time for each experiment of 23 h. The ^13^C^ζ^–^15^N^ε^ HSQC (Fig. 4a) spectrum was acquired as a 512 × 48 complex matrix with spectral widths of 11 ppm (^13^C) and 10 ppm (^15^N). 128 scans were collected for each *t*_1_ increment with a recycle delay of 3 s resulting in a total experiment time of 12 h. The 3D ^13^C^ζ^–^15^N^ε^–^15^N^η^ experiment (Fig. 4b) was acquired as a 1024 × 32 × 32 complex cube with spectral widths of 40 ppm (^13^C) and 10 ppm (^15^N^ε^ and ^15^N^η^). 16 scans were collected for each *t*_1_/*t*_2_ increment with a recycle delay of 3.5 s resulting in a total experiment time of 71 h. The spectra in Fig. 5 were collected as above using pulse sequences modified to include ^2^H decoupling in the indirect dimension. The ^2^H signal of the lock solvent was preserved by flanking the decoupling sequence with a pair of opposite phase ^2^H pulses (*y,− y*), both of which were orthogonal to the decoupling field (*x*). NMR data was processed using NMRPipe (Delaglio et al. 1995) and subsequently analysed using the Analysis module of the CCPNMR package (Vranken et al. 2005).

## Results and discussion

The conventional approach to probe the chemical shift of an amine or an amide ^15^N within a biomolecule involves the collection of ^1^H–^15^N correlation experiments, which make use of the ^1^ *J*_HN_ scalar coupling between the ^15^N nucleus and the directly bound proton (Bodenhausen and Ruben 1980). This inverse-type experiment employs ^1^H detection, which affords high sensitivity as well as an additional chemical shift dimension that reduces spectral crowding and subsequent signal overlap. However, for arginine side-chains, two chemical exchange processes lead to severe line broadenings of the ^1^H–^15^N correlations, in particular at physiological pH and at ambient temperature. Firstly, the directly bound ^1^H^ε^ and ^1^H^η^ protons of the guanidinium group undergo a rapid chemical exchange with the solvent, which lead to a line broadening in the ^1^H dimension and an attenuation of the signals in the NMR experiment. In samples prepared at neutral and high pH, the line broadening and loss of signal intensity is so severe that it often prevents a detection of the signal, unless the proton in question is involved in a strong hydrogen bond (Zeymer et al. 2016). In samples prepared at lower pH (< 6.5), the exchange is sufficiently slowed so that ^1^H^ε^–^15^N^ε^ HSQC correlation spectra can be obtained (Morgan et al. 1999; Trbovic et al. 2009; Iwahara and Clore 2006). For example, for the 19 kDa L99A mutant of T4 Lysozyme (T4L99A) at pH 5.5 and at 298 K all of the 13 arginine side-chains can be observed in ^1^H^ε^–^15^N^ε^ HSQC correlation spectra (Fig. 1b, ϖ_1_(^15^N) ~ 85 ppm). Nonetheless, even with the favourable ^1^H exchange conditions, the ^1^H^η^–^15^N^η^ correlations largely remain significantly broadened and of low intensity (Fig. 1b, ϖ_1_(^15^N) ~ 71 ppm). This is a manifestation of a second exchange process resulting from the restricted rotation about the C^ζ^–N^ε^ bond.

With adaptations to a recently published NMR experiment (Gerecht et al. 2017; Werbeck et al. 2013), carbon-detected ^13^C^ζ^–^15^N^η^ HSQC can be employed to overcome the line broadenings associated with the exchange of the ^1^H^η^ protons with bulk solvent (Yoshimura et al. 2017). In the previous ^13^C^ζ^–^15^N^ε^ HSQC experiment (Werbeck et al. 2013), ^13^C^ζ^ equilibrium magnetisation is selectively excited using an Eburp-2 pulse (Geen and Freeman 1991) and transferred to the two-spin order longitudinal spin density matrix element, $2C_{z}^{\zeta }N_{z}^{\varepsilon }$, using an INEPT (Morris and Freeman 1979) sequence of length 1/(2*J*_CN_) (approx. 25 ms) with selective inversion of ^15^N^ε^. Anti-phase transverse $2C_{z}^{\zeta }N_{{{{x,y}}}}^{\varepsilon }$ magnetisation is subsequently evolved and transferred back to transverse $C_{x}^{\zeta }$ for detection. Two changes were made in order to obtain the ^13^C^ζ^–^15^N^η^ HSQC spectra: (1) change the inversion pulse in the INEPT blocks to be selective for ^15^N^η^ and (2) change the length of the INEPT to 1/(4*J*_CN_). Thus, after the first INEPT, the resulting magnetisation of interest is proportional to $2C_{z}^{\zeta }N_{z}^{{{{{\eta}1}}}}+2C_{z}^{\zeta }N_{z}^{{{{{\eta}2}}}}$. As the ^13^C^ζ^–^15^N^η^ HSQC experiment relies on the chemical shift evolution of ^13^C^ζ^ and ^15^N^η^ as well as the ^1^ *J*_CN_ scalar coupling between them, the exchange of ^1^H^η^ with the bulk solvent does not affect the obtained spectrum. It should be noted that because the experiment is based on ^13^C-excitation and ^13^C-detection there is an intrinsic sensitivity penalty owing to the lower gyromagnetic ratio of ^13^C compared to ^1^H. However, in the case of ^15^N^η^, this is outweighed by the elimination of the exchange with the bulk solvent, thus resulting in a clear improvement over the ^1^H–^15^N spectrum (Fig. 1c). In spite of that many of the ^13^C^ζ^–^15^N^η^ correlations are substantially broadened in the ^15^N dimension and consequently overlapped.

Owing in part to the ability to form salt-bridges with negatively charged side-chains such as aspartic and glutamic acids, a range of C^ζ^–N^ε^ bond rotational rates are typically observed for arginine residues in proteins (Nieto et al. 1997; Gerecht et al. 2017). How these rates affect the NMR spectra depends on both the rate of rotational exchange and the absolute chemical shift difference between the two exchanging sites; in this case ^15^N^η1^ and ^15^N^η2^. In the slow-exchange regime (*k*_ex_ $\ll \; {\big |}$ω(^15^N^η1^) − ω(^15^N^η2^)${\big |}$), and for arginine side-chains involved in strong hydrogen bonds or salt bridges, a sharp signal is observed for each of the two ^15^N^η^ (*e.g*. R52, R148, R95 in T4L99A), whilst in the fast-exchange regime (*k*_ex_ $\gg \; {\big |}$ω(^15^N^η1^) − ω(^15^N^η2^)${\big |}$) a single sharp signal is observed at the average chemical shift (e.g. free arginine at elevated temperature). In the intermediate exchange regime, as the rate of exchange approaches the chemical shift difference (*k*_ex_ ≈ ${\big |}$ω(^15^N^η1^) − ω(^15^N^η2^)${\big |}$), the signals coalesce and result in a broad resonance that is often at the limit of detection. This exchange-broadening is apparent for R14 and several signals around 157.0 ppm (^13^C) in T4L99A (Fig. 1c). An arginine single-quantum ^13^C^ζ^–^15^N^ε/η^ experiment has been published recently (Yoshimura et al. 2017), where the effect of chemical exchange is minimised by a combination of cross-polarisation and ^13^C detection. Although the cross-polarisation quenches the exchange-broadening caused by the exchange with the solvent and the rotation about the C^ζ^–N^ε^ bond during transfer steps, the resulting single-quantum ^13^C^ζ^–^15^N^η^ spectrum still suffers from significant overlap. As described below a double-quantum coherence can be created that is insensitive to the rotation about the C^ζ^–N^ε^ bond and thus unaffected by the line broadening resulting from this exchange process.

### A ^15^N^η^ double-quantum experiment

The basic crux of our approach to characterise ^15^N^η^ chemical shifts is to create a double-quantum coherence that is insensitive to the rotation about the C^ζ^–N^ε^ bond. Firstly, it is noted that the nature of the exchange between ^15^N^η1^ and ^15^N^η2^ means that the population of the two exchanging sites is identical. Secondly, the double-quantum coherences $4C_{z}^{\zeta }N_{+}^{{{{{\eta}1}}}}N_{+}^{{{{{\eta}2}}}}$ and $4C_{z}^{\zeta }N_{ - }^{{{{{\eta}1}}}}N_{ - }^{{{{{\eta}2}}}}$ evolve under the free precession Hamiltonian with frequencies of ±(ω(^15^N^η1^) + ω(^15^N^η2^)), respectively, where $C_{i}^{\zeta }$, $N_{i}^{{\eta}1}$ and $N_{i}^{{{{{\eta}2}}}}$ denote ^13^C^ζ, 15^N^η1^ and ^15^N^η2^ standard spin density operator matrix elements, respectively (Sørensen et al. 1984). A chemical exchange that interchanges ^15^N^η1^ and ^15^N^η2^, therefore leaves the double-quantum precession frequencies unchanged. Consequently, the rotation about the C^ζ^–N^ε^ bond does not affect the evolution of the double-quantum coherences $4C_{z}^{\zeta }N_{+}^{{{{{\eta}1}}}}N_{+}^{{{{{\eta}2}}}}$ and $4C_{z}^{\zeta }N_{ - }^{{{{{\eta}1}}}}N_{ - }^{{{{{\eta}2}}}}$, and thus no exchange-broadening arising from this rotation is expected to be observed.

The pulse sequence that was developed here to evolve the double-quantum coherences $4C_{z}^{\zeta }N_{+}^{{{{{\eta}1}}}}N_{+}^{{{{{\eta}2}}}}$ and $4C_{z}^{\zeta }N_{ - }^{{{{{\eta}1}}}}N_{ - }^{{{{{\eta}2}}}}$ of arginine side-chains is shown in Fig. 2a. Briefly, magnetisation is selectively transferred from ^13^C^ζ^ to ^15^N^η^ *via* the one bond scalar coupling ^1^ *J*_CN_ using an INEPT element that incorporates selective ^13^C^ζ^ excitation and a selective ^15^N^η^ inversion pulse. A density element proportional to the three-spin order longitudinal density element $4C_{z}^{\zeta }N_{z}^{{{{{\eta}1}}}}N_{z}^{{{{{\eta}2}}}}$ is obtained at point *a* by allowing $C_{y}^{\zeta }$ to evolve for 2Δ = 1/(2*J*_CN_) under the scalar coupling Hamiltonian. Subsequently a ${\text{90}}_{{\upphi {{2~=~{x}}}}}^{\circ }$ ^15^N pulse generates the multiple-quantum coherence $4C_{z}^{\zeta }N_{y}^{{{{{\eta}1}}}}N_{y}^{{{{{\eta}2}}}}=Z{Q_x} - D{Q_x}$, where:

1 $$D{Q_{{x}}}={\text{0.5(4}}C_{z}^{\zeta }N_{x}^{{{{{\eta}1}}}}N_{x}^{{{{{\eta}2}}}} - {\text{~4}}C_{z}^{\zeta }N_{y}^{{{{{\eta}1}}}}N_{y}^{{{{{\eta}2}}}})$$

2 $$Z{Q_{{x}}}={\text{0.5}}(4_{z}^{\zeta }N_{x}^{{{{{\eta}1}}}}N_{x}^{{{{{\eta}2}}}}+4C_{z}^{\zeta }N_{y}^{{{{{\eta}1}}}}N_{y}^{{{{{\eta}2}}}})$$

The double-quantum component is selected by phase-cycling the ^15^N excitation pulse (*x,y*) with a concomitant inversion of the receiver phase. Subsequently the selected double-quantum coherence, $D{Q_{{x}}}=0.5{\text{(4}}C_{z}^{\zeta }N_{x}^{{{{{\eta}1}}}}N_{x}^{{{{{\eta}2}}}} - {\text{4}}C_{z}^{\zeta }N_{y}^{{{{{\eta}1}}}}N_{y}^{{{{{\eta}2}}}})$, is allowed to evolve between *a* and *b* during the variable delay, *t*_1_, where the evolutions under the one-bond ^1^H–^15^N scalar couplings are suppressed with a ^1^H WALTZ decoupling scheme (André et al. 2007; Shaka et al. 1983). Coupling to the ^13^C^ζ^ nucleus is refocused by a 180° ^13^C pulse in the middle of the *t*_1_ period. The evolution proceeds according to,

3 $$ DQ_{x} \xrightarrow{{(\Omega _{{{\text{N}}\eta 1}} N_{z}^{{\eta 1}} + \Omega _{{{\text{N}}\eta 2}} N_{z}^{{\eta 2}} )t_{1} }}DQ_{x} \cos \left[ {\left( {\Omega _{{{\text{N}}\eta 1}} + \Omega _{{{\text{N}}\eta 2}} } \right)t_{1} } \right]~ + ~DQ_{y} \sin \left[ {\left( {\Omega _{{{\text{N}}\eta 1}} + \Omega _{{{\text{N}}\eta 2}} } \right)t_{1} } \right] $$

where the *DQ*_x_ coherence evolves with the sum of the two underlying ^15^N frequencies (Ω_Nη1_ + Ω_Nη2_) during *t*_1_, and $DQ_{{{y}}} = 0.5(4C_{z}^{\zeta } N_{x}^{{\eta 1}} N_{y}^{{\eta 2}} + ~4C_{z}^{\zeta } N_{y}^{{\eta 1}} N_{x}^{{\eta 2}} )$. The ${\text{90}}_{x}^{\circ }$ ^15^N pulse followed by the gradient pulse g4 at point *b* selects for the $4C_{z}^{\zeta } N_{y}^{{\eta 1}} N_{y}^{{\eta 2}}$ component of the double-quantum coherence, which is transferred back to transverse in-phase carbon magnetisation, $C_{y}^{\zeta }$, for detection *via* a retro-INEPT between *b* and *c*, again incorporating a selective ^15^N^η^ inversion pulse. Frequency discrimination in the indirect dimension is achieved by incrementing the ${\text{90}}_{{\upphi {\text{2}}}}^{\circ }$ ^15^N pulse by 45° (Bax et al. 1981). It is important to note that in order to eliminate the effect of the exchange between the two ^15^N^η^, the experiment has been designed such that the magnetisation of interest does not at any point exist as transverse single-quantum ^15^N magnetisation.

Two-dimensional Fourier transformation of the interferogram results in a signal for each arginine residue with the ^13^C^ζ^ frequency along the direct dimension and the sum of the two coupled ^15^N^η^ frequencies, ${\Omega _{{{\text{N}{\eta}1}}}}+{{{{\Omega}}}_{{{\text{N}{\eta}2}}}},$ along the indirect dimension. The exchange of the two ^15^N^η^ sites with one another has no effect on the double-quantum frequency and thus the broad, featureless signals in the ^13^C^ζ^–^15^N^η^ HSQC are rendered substantially sharper (Fig. 2b). In the spectrum in Fig. 2b, a single peak is observed for each arginine residue and the data is processed such that the indirect chemical shift reflects the average of the two contributing ^15^N^η^ nuclei. The double-quantum experiment is particularly useful to probe flexible arginine side chains, where in the case of T4L99A all eight signals around 157.0 ppm (^13^C) are well resolved. A disadvantage of the double-quantum experiment compared to the single-quantum experiment is the faster (ca. twofold) transverse relaxation during the indirect chemical shift evolution period, since the spin density matrix elements evolved, $4C_{z}^{\zeta } N_{{x,y}}^{{\eta 1}} N_{{x,y}}^{{\eta 2}} ~$, are transverse with respect to both ^15^N^η^ nuclei, which in turn relax with the two directly bound protons. The faster relaxation, which leads to lower signal-to-noise, only becomes significant for arginine side-chains that are rigid, for example, R95 and R148 (Werbeck et al. 2013) (Fig. 2b). However, the rigid side-chains are typically less affected by the exchange process and so the data obtained from the ^13^C^ζ^–^15^N^η^ HSQC experiment is often useful for these residues (Fig. 1c). The substantially better resolution provided by the double-quantum experiment adequately compensates for the associated loss of signal for less rigid residues.

The rapid transverse relaxation of the ^15^N^η^ nuclei of rigid residues in medium-to-large proteins can be mitigated by preparing the sample in a 100% D_2_O buffer. Substitution of the ^1^H^η^ protons with deuterium leads to slower ^15^N^η^ transverse relaxation and thus sharper lines in the indirect dimension of the double-quantum experiment. However, the longitudinal relaxation time of the ^13^C^ζ^ nucleus also increases, which limits the permitted recycle rate of the experiment and increases the overall acquisition time. Whilst we have not observed a significant sensitivity gain per unit time using a 100% D_2_O buffer, recording spectra in 100% D_2_O could be useful in applications where experimental time is not a concern. Such an approach may enable the study of even larger proteins.

### A route for chemical shift assignments of the ^13^C^ζ^–^15^N^η^(DQ) spectrum

**Fig. 3:**
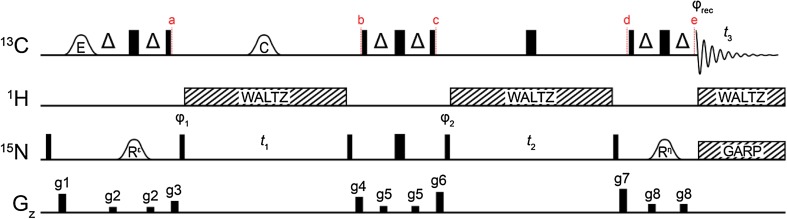
Pulse sequence to obtain the intra-residue correlation between ^15^N^ε^ and ^15^N^η^ double-quantum chemical shifts. The carrier positions are ^13^C: 156 ppm, ^15^N: 84 ppm (R^ε^), 78 ppm (square 180° and decoupling), 71 ppm (R^η^) and ^1^H: 7 ppm. Narrow and wide bars represent 90° and 180° pulses respectively and are applied at maximum power. The delay Δ is 1/(4*J*_CN_) = 12.5 ms. Shaped pulses are represented by bell shapes with letters indicating the shape of the pulse [E: E-BURP-2, R^ε^: ^15^N^ε^ selective RE-BURP, R^η^: ^15^N^η^ RE-BURP^41^, C: smoothed CHIRP (Ermakov et al. 1993)] and are applied with durations of 1.5 ms (E), 6 ms (R^ε^, R^η^) and 500 μs (C) at 11.74 T. Pulses are *x* phase unless stated otherwise. The phase cycle used is φ_1_: *x, − x*, φ_2_: 2(*x*), 2(*y*), 2(*− x*), 2(*− y*), φ_rec_: *x*, 2(*− x*), *x*. Decoupling sequences are represented by striped boxes indicating the type of decoupling: WALTZ64 (Zhou et al. 2007) (4 kHz), GARP4 (Shaka et al. 1985) (0.7 kHz). Gradient pulses of 1 ms are represented by black rectangles and applied with strengths of g1: 19.8 G/cm, g2: 5.9 G/cm, g3: 12.3 G/cm, g4: 16.6 G/cm, g5: 7.0 G/cm, g6: 21.9 G/cm, g7: 25.2 G/cm, g8: 9.1 G/cm

**Fig. 4:**
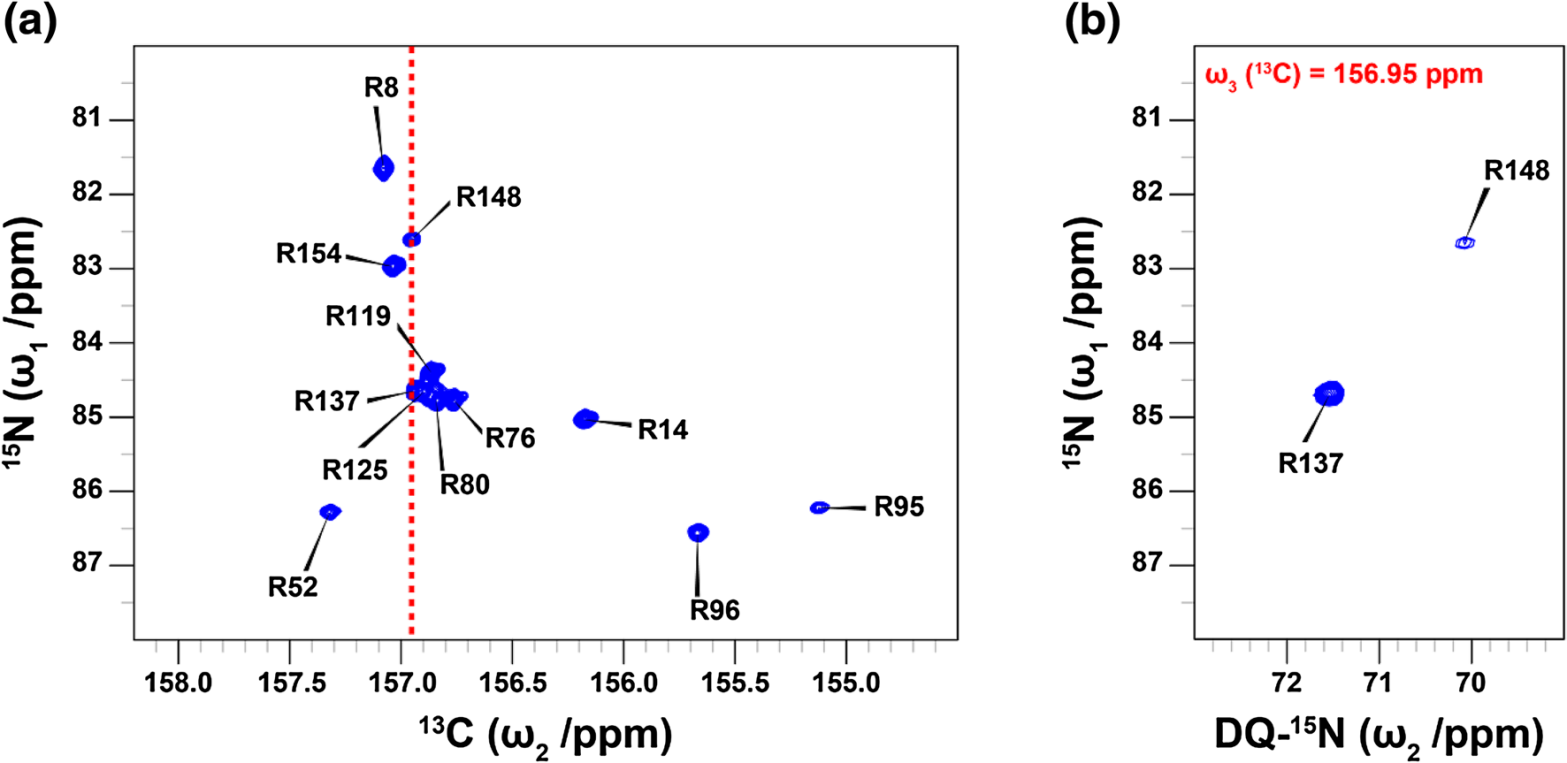
**a** ^13^C^ζ^–^15^N^ε^ HSQC spectrum of T4L99A recorded at 16.4 T. The overlap of R137 and R148 in the ^13^C dimension is highlighted by red dashed line. This ambiguity hampers the chemical shift assignment of the double-quantum spectrum in Fig. 2b. Chemical shift assignments are taken from Werbeck et al. (2013). **b** ^15^N^ε^–^15^N^η^(DQ) 2D-plane extracted at ^13^C^ζ^ = 156.95 ppm (red line in **a**) from the 3D experiment (Fig. 3) recorded at 11.74 T. The 3D spectrum allows unambiguous chemical shift assignment of the ^15^N^η^ double-quantum spectrum in Fig. 2b

In favourable circumstances, an existing ^13^C^ζ^–^15^N^ε^ assignment can be transferred to the ^13^C^ζ^–^15^N^η^(DQ) spectrum based on the ^13^C^ζ^ chemical shift alone. However spectral overlap of the arginine ^13^C^ζ^ is not uncommon in even modestly-sized proteins. The ^15^N^η^ double-quantum experiment described above can be embedded within the existing ^13^C^ζ^–^15^N^ε^ HSQC sequence (Werbeck et al. 2013) to provide a three-dimensional experiment for chemical shift assignment; Fig. 3. Briefly, magnetisation proportional to $2C_{z}^{\zeta }N_{z}^{\varepsilon }$ is generated *via* an INEPT block with ^13^C^ζ^ and ^15^N^ε^ selective pulses. This magnetisation is then allowed to evolve during the first chemical shift evolution period *t*_1_, between *a* and *b*, to encode the ^15^N^ε^ chemical shift. One-bond scalar couplings to ^1^H^ε^ and ^13^C^ζ/δ^ are refocused with a ^1^H WALTZ decoupling scheme and a ^13^C adiabatic inversion pulse, respectively. A second INEPT block, between *b* and *c*, with a *non-selective* high power 180° ^15^N pulse, cleanly converts the $2C_{z}^{\zeta }N_{z}^{\varepsilon }$ longitudinal two-spin order element to the three-spin order element $4C_{z}^{\zeta } N_{z}^{{\eta 1}} N_{z}^{{\eta 2}}$. As shown above, the double-quantum component is selected with a phase-cycle and allowed to evolve, with ^1^H and ^13^C decoupling, during the second chemical shift evolution period *t*_2_. Finally, the magnetisation is returned to in-phase carbon, $C_{y}^{\zeta }$, for detection between *d* and *e*, using a third INEPT block that is selective only for ^15^N^η^. Figure 4 demonstrates how the resulting three-dimensional dataset is used to unambiguously assign the ^15^N^η^ resonances R137 and R148 in T4L99A, both of which have a ^13^C^ζ^ frequency of 156.95 ppm.

### Accessing small and residue-specific chemical shift perturbations

The observed chemical shift of a particular nucleus in an NMR experiment is very sensitive to the local molecular environment. For many years, localised chemical shift changes in NMR spectra have been used to measure side-chain pK_a_s (Wang et al. 1996; Tollinger et al. 2002), investigate ligand binding (Williamson 2013) and assess protein folding (Calzolai and Zahn 2003). More recently, the magnitude of the deuterium isotope shift of lysine amines (Tomlinson et al. 2009; Williamson et al. 2013) has been used to infer the presence of solution-state salt-bridges in proteins. The technique relies on the detection of a small ^15^N chemical shift difference observed for an amine when one or more of the bound ^1^H are exchanged with deuterium (D) and thus highly resolved NMR spectra are essential. Arginine is very well suited to the formation of salt bridges and the deuterium isotope shift of the terminal –N^η^H_2_ amines is likely to be a useful parameter to characterise salt-bridge formation in solution. It has previously not been possible to measure these potentially very small and useful isotope shifts using standard proton-detected NMR experiments due to the shortcomings of these experiments described above. However, the double-quantum experiment presented above opens up a possible route for determining the isotope shifts, as well as other chemical shift perturbations, of the –N^η^H_2_ amines of arginine side-chains.

**Fig. 5:**
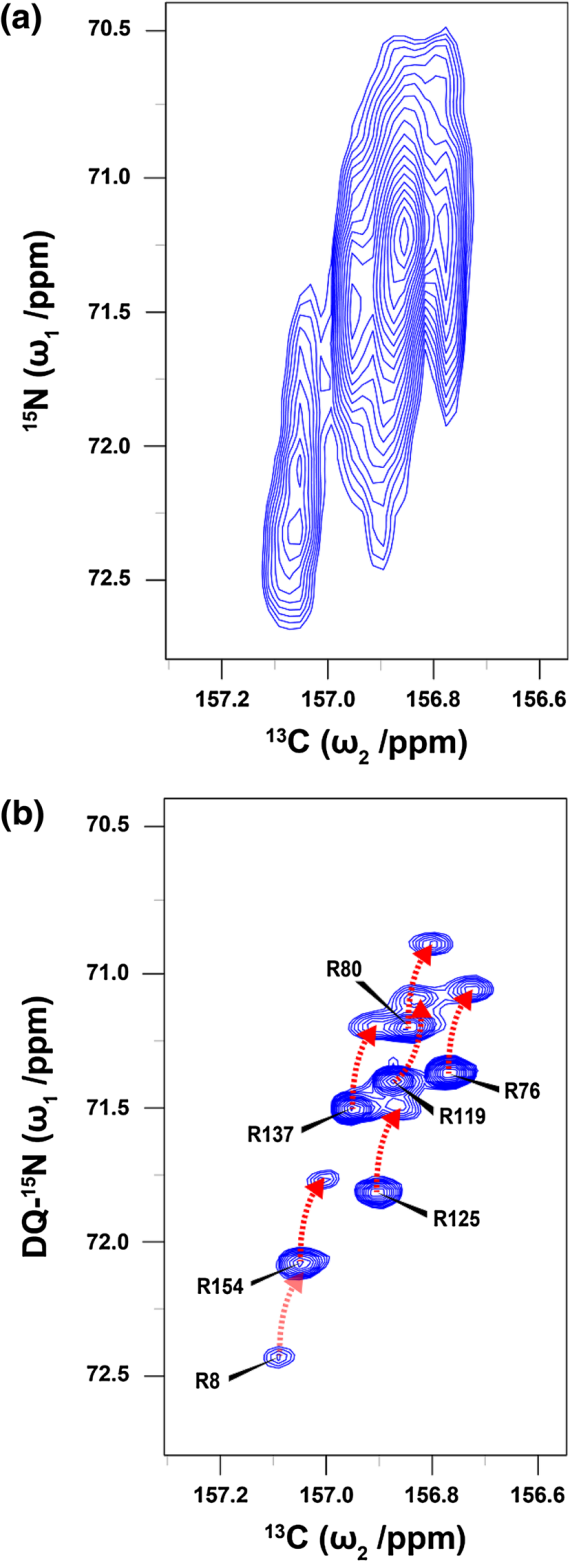
**a** Extreme signal overlap observed in the ^13^C^ζ^–^15^N^η^ HSQC spectrum of T4L99A recorded at 18.8 T in a buffer containing 10 vol.% D_2_O. **b** ^13^C^ζ^–^15^N^η^ HDQC spectrum recorded on the same sample. The resolution is high enough to quantify the residue-specific deuterium isotope shifts of all but one of the arginine side-chain amines contributing to the very broad signal in (**a**). The marked peaks indicate the –^15^N^η^_2_H_4_ isotopomer and the red arrow indicates the location of the corresponding –^15^N^η^_2_H_3_D isotopomer. Only two peaks are overlapped in this spectrum, that is –^15^N^η^_2_H_3_D signal for R8 overlaps with the –^15^N^η^_2_H_4_ signal of R154. The isotope shifts of the remaining residues are easily identified from this data. The pulse sequences used to obtain the spectra in **a** and **b** have been modified to include ^2^H decoupling (WALTZ16, 1 kHz) during the indirect evolution period

The − ^15^N^η^H_*j*_D_2−*j*_, *j* = 0,1,2 isotopomers of arginine side-chains are generated by dissolving the protein sample in a buffer containing a suitable percentage of D_2_O (10–30 vol.%). The conventional carbon-detected ^13^C^ζ^–^15^N^η^ HSQC experiment is applicable to measure the deuterium isotope shift for arginine side-chains that are slowly exchanging about the C^ζ^–N^ε^ bond and give rise to two separate ^13^C^ζ^–^15^N^η^ resonances, for example R96 in T4L99A. Unfortunately, the addition of D_2_O increases the number of signals observed in an already overcrowded spectral region. For a significant number of residues in T4L99A, intermediate exchange of the two ^15^N^η^ sites combined with spectral overlap of isotopomers makes a quantification of residue-specific isotope shifts nearly impossible using the ^13^C^ζ^–^15^N^η^ HSQC experiment (Fig. 5a). The increased resolution afforded by the double-quantum experiment means that it is very well suited for the measurement of small chemical shift perturbations, such as the isotope shift, for the vast majority of arginine residues; particularly those that are in intermediate exchange regimes (Fig. 5b; Table 1). Even at a temperature of 278 K, where the rotational correlation time of T4L99A approaches 20 ns, the majority of these flexible arginine side-chains are still well resolved (Figure S1).

**Table 1:** Deuterium isotope shifts measured for selected arginine side-chains in T4L99A

Residue	^2^Δ^13^C^ξ^ (N^η^–H)/ppm	^1^Δ^15^N^η^ (H)/ppm
R8	*ND* ^a^	*ND* ^a^
R76	0.044 ± 0.001	0.312 ± 0.001
R80	0.046 ± 0.002	0.313 ± 0.001
R119	0.040 ± 0.002	0.309 ± 0.002
R125	0.039 + 0.001	0.322 ± 0.005
R137	0.045 ± 0.001	0.308 ± 0.004
R154	0.045 ± 0.003	0.312 ± 0.004
Free Arg^b^	0.042 ± 0.003	0.307 ± 0.006

^a^In the case of R8, the signal corresponding to the singly-deuterated isotopomer overlaps with the protonated signal of R154 and thus hampers an accurate determination of the shift

^b^Values for free arginine were obtained on a 25 mM sample of [^13^C,^15^N]-arginine hydrochloride in phosphate buffer at pH 5.5 containing 50 vol.% D_2_O. Uncertainties were obtained from duplicate measurements

The obtained isotope shifts for the flexible arginine residues of T4L99A correlate very closely with the value measured for free arginine. This suggests that the solvent-exposed and flexible residues are not involved in any significant interactions. It should be noted that due to the nature of the double-quantum experiment, the individual isotope shift of ^15^N^η1^ and ^15^N^η2^ cannot be distinguished using this sequence. Nonetheless, an important experimental parameter that reports on interactions of the arginine side-chain can be obtained. Further NMR experiments combined with theoretical approaches to further characterise the hydrogen-bonding and salt-bridging behaviour of arginine residues are on-going.

## Summary and conclusion

In summary, we presented pulse schemes to characterise arginine side-chain ^15^N^η^ amines in solution. The preparation and subsequent evolution of a double-quantum ^15^N^η^ coherence eliminates the line broadenings associated with slow-to-intermediate rotation about the C^ζ^–N^ε^ partial double bond thus leading to dramatically sharpened peaks in the NMR spectrum. The double-quantum experiment is complementary to the ^13^C^ζ^–^15^N^η^ single-quantum experiment because the double-quantum experiment is ideally suited to characterise arginine side-chains whose ^13^C^ζ^–^15^N^η^ single-quantum resonances are severely broadened because of exchange, whilst the single-quantum experiment provides (^15^N^η1^,^15^N^η2^) site-specific information for the well-resolved resonances. An application to the 19 kDa T4L99A protein demonstrated the strengths of the double-quantum experiment and allowed the quantification of small deuterium isotope shifts to provide information on the interactions of the arginine side-chain guanidinium group. The magnitude of the detected isotope shifts showcases the high resolution of this experiment and suggests its potential application to many other applications where residue-specific chemical shift perturbations are of interest. The presented experiments add to a growing list of methods for characterising functional protein side-chains, which ultimately will allow a quantification of the structure, dynamics, and interactions of side-chains in solution to a level where their specific contribution to enzymatic function and protein interactions can be elucidated.

## Electronic supplementary material

Below is the link to the electronic supplementary material.

Supplementary material 1 (PDF 445 KB)

## References

[CR1] Andersson P, Gsell B, Wipf B, Senn H, Otting G (1998). HMQC and HSQC experiments with water flip-back optimized for large proteins. J Biomol NMR.

[CR2] André I, Linse S, Mulder FAA (2007). Residue-specific pKa determination of lysine and arginine side chains by indirect ^15^N and ^13^C NMR spectroscopy: application to apo calmodulin. J Am Chem Soc.

[CR3] Bax A, Freeman R, Frenkiel TA, Levitt MH (1981). Assignment of carbon-13 NMR spectra via double-quantum coherence. J Magn Reson.

[CR4] Bodenhausen G, Ruben DJ (1980). Natural abundance nitrogen-15 NMR by enhanced heteronuclear spectroscopy. Chem Phys Lett.

[CR5] Borders CL, Broadwater JA, Bekeny PA, Salmon JE, Lee AS, Eldridge AM, Pett VB (2008). A structural role for arginine in proteins: multiple hydrogen bonds to backbone carbonyl oxygens. Protein Sci.

[CR6] Calzolai L, Zahn R (2003). Influence of pH on NMR structure and stability of the human prion protein globular domain. J Biol Chem.

[CR7] Carr HY, Purcell EM (1954). Effects of diffusion on free precession in nuclear magnetic resonance experiments. Phys Rev.

[CR8] Casey AK, Hicks MA, Johnson JL, Babbitt PC, Frantom PA (2014). Mechanistic and bioinformatic investigation of a conserved active site helix in α-isopropylmalate synthase from *Mycobacterium tuberculosis*, a member of the DRE-TIM metallolyase superfamily. Biochemistry.

[CR9] Crowley PB, Golovin A (2005). Cation-π interactions in protein-protein interfaces. Proteins Struct Funct Bioinforma.

[CR10] Delaglio F, Grzesiek S, Vuister G, Zhu G, Pfeifer J, Bax A (1995). NMRPipe: a multidimensional spectral processing system based on UNIX pipes. J Biomol NMR.

[CR11] Ermakov VL, Bohlen JM, Bodenhausen G (1993). Improved schemes for refocusing with frequency-modulated chirp pulses. J Magn Reson Ser A.

[CR12] Farber PJ, Mittermaier A (2015). Relaxation dispersion NMR spectroscopy for the study of protein allostery. Biophys Rev.

[CR13] Fitch CA, Platzer G, Okon M, Garcia-Moreno EB, McIntosh LP (2015). Arginine: its pKa value revisited. Protein Sci.

[CR14] Friedt J, Leavens FMV, Mercier E, Wieden H-J, Kothe U (2014). An arginine-aspartate network in the active site of bacterial TruB is critical for catalyzing pseudouridine formation. Nucleic Acids Res.

[CR15] Gargaro AR, Frenkiel TA, Nieto PM, Birdsall B, Polshakov VI, Morgan WD, Feeney J (1996). NMR detection of arginine-ligand interactions in complexes of Lactobacillus casei dihydrofolate reductase. Eur J Biochem.

[CR16] Geen H, Freeman R (1991). Band-selective radiofrequency pulses. J Magn Reson.

[CR17] Gerecht K, Figueiredo AM, Hansen DF (2017). Determining rotational dynamics of the guanidino group of arginine side chains in proteins by carbon-detected NMR. Chem Commun.

[CR18] Goldschen-Ohm MP, Wagner DA, Jones MV (2011). Three arginines in the GABAA receptor binding pocket have distinct roles in the formation and stability of agonist- versus antagonist-bound complexes. Mol Pharmacol.

[CR19] Hansen PE (2000). Isotope effects on chemical shifts of proteins and peptides. Magn Reson Chem.

[CR20] Hansen DF, Led JJ (2003). Implications of using approximate Bloch–McConnell equations in NMR analyses of chemically exchanging systems: application to the electron self-exchange of plastocyanin. J Magn Reson.

[CR21] Hansen DF, Vallurupalli P, Kay LE (2008). An improved 15N relaxation dispersion experiment for the measurement of millisecond time-scale dynamics in proteins. J Phys Chem B.

[CR22] Harms MJ, Schlessman JL, Sue GR, Garcia-Moreno EB (2011). Arginine residues at internal positions in a protein are always charged. Proc Natl Acad Sci USA.

[CR23] Henry GD, Sykes BD (1995). Determination of the rotational dynamics and pH dependence of the hydrogen exchange rates of the arginine guanidino group using NMR spectroscopy. J Biomol NMR.

[CR24] Iwahara J, Clore GM (2006). Sensitivity improvement for correlations involving arginine side-chain Ne/He resonances in multi-dimensional NMR experiments using broadband ^15^N 180° pulses. J Biomol NMR.

[CR25] Kleckner IR, Foster MP (2011). An introduction to NMR-based approaches for measuring protein dynamics. Biochim Biophys Acta Proteins Proteomics.

[CR26] Korzhnev DM, Kay LE (2008). Probing invisible, low-populated states of protein molecules by relaxation dispersion nmr spectroscopy: an application to protein folding. Acc Chem Res.

[CR27] McConnell HM (1958). Reaction rates by nuclear magnetic resonance. J Chem Phys.

[CR28] Morgan WD, Birdsall B, Nieto PM, Gargaro AR, Feeney J (1999). ^1^H/^15^N HSQC NMR studies of ligand carboxylate group interactions with arginine residues in complexes of brodimoprim analogues and lactobacillus casei dihydrofolate reductase. Biochemistry.

[CR29] Morris GA, Freeman R (1979). Enhancement of nuclear magnetic resonance signals by polarization transfer. J Am Chem Soc.

[CR30] Nieto PM, Birdsall B, Morgan WD, Frenkiel TA, Gargaro AR, Feeney J (1997). Correlated bond rotations in interactions of arginine residues with ligand carboxylate groups in protein ligand complexes. FEBS Lett.

[CR31] Orekhov VY, Korzhnev DM, Kay LE (2004). Double- and zero-quantum NMR relaxation dispersion experiments sampling millisecond time scale dynamics in proteins. J Am Chem Soc.

[CR32] Palmer AG, Massi F (2006). Characterization of the dynamics of biomacromolecules using rotating-frame spin relaxation NMR spectroscopy. Chem Rev.

[CR33] Palmer AG (2014). Chemical exchange in biomacromolecules: Past, present, and future. J Magn Reson.

[CR34] Pervushin KV, Wider G, Riek R, Wuthrich K (1999). The 3D NOESY-[^1^H,^15^N,^1^H]-ZQ-TROSY NMR experiment with diagonal peak suppression. Proc Natl Acad Sci USA.

[CR35] Rohs R, Jin X, West SM, Joshi R, Honig B, Mann RS (2010). Origins of specificity in protein-DNA recognition. Annu Rev Biochem.

[CR36] Shaka AJ, Keeler J, Frenkiel T, Freeman R (1983). An improved sequence for broadband decoupling: WALTZ-16. J Magn Reson.

[CR37] Shaka AJ, Barker PB, Freeman R (1985). Computer-optimized decoupling scheme for wideband applications and low-level operation. J Magn Reson.

[CR38] Sørensen OW, Eich GW, Levitt MH, Bodenhausen G, Ernst RR (1984). Product operator formalism for the description of NMR pulse experiments. Prog Nucl Magn Reson Spectrosc.

[CR39] Tollinger M, Forman-Kay JD, Kay LE (2002). Measurement of side-chain carboxyl pKa values of glutamate and aspartate residues in an unfolded protein by multinuclear NMR spectroscopy. J Am Chem Soc.

[CR40] Tomlinson JH, Ullah S, Hansen PE, Williamson MP (2009). Characterization of salt bridges to lysines in the protein G B1 domain. J Am Chem Soc.

[CR41] Trbovic N, Cho J-H, Abel R, Friesner RA, Rance M, Palmer AG (2009). Protein side-chain dynamics and residual conformational entropy. J Am Chem Soc.

[CR42] Vallurupalli P, Hansen DF, Lundström P, Kay LE (2009). CPMG relaxation dispersion NMR experiments measuring glycine 1Hα and 13Cα chemical shifts in the ‘invisible’ excited states of proteins. J Biomol NMR.

[CR43] Vranken WF, Boucher W, Stevens TJ, Fogh RH, Pajon A, Llinas M, Ulrich EL, Markley JL, Ionides J, Laue ED (2005). The CCPN data model for NMR spectroscopy: development of a software pipeline. Proteins Struct Funct Bioinforma.

[CR44] Wang Y-X, Freedberg DI, Yamazaki T, Wingfield PT, Stahl SJ, Kaufman JD, Kiso Y, Torchia DA (1996). Solution NMR evidence that the HIV-1 protease catalytic aspartyl groups have different ionization states in the complex formed with the asymmetric drug KNI-272 †. Biochemistry.

[CR45] Werbeck ND, Kirkpatrick J, Hansen DF (2013). Probing arginine side-chains and their dynamics with carbon-detected nmr spectroscopy: application to the 42 kDa human histone deacetylase 8 at high pH. Angew Chemie Int Ed.

[CR46] Williamson MP (2013). Using chemical shift perturbation to characterise ligand binding. Prog Nucl Magn Reson Spectrosc.

[CR47] Williamson MP, Hounslow AM, Ford J, Fowler K, Hebditch M, Hansen PE (2013). Detection of salt bridges to lysines in solution in barnase. Chem Commun.

[CR48] Yamazaki T, Pascal SM, Singer AU, Forman-Kay JD, Kay LE (1995). NMR pulse schemes for the sequence-specific assignment of arginine guanidino ^15^N and ^1^H chemical shifts in proteins. J Am Chem Soc.

[CR49] Yoshimura Y, Oktaviani NA, Yonezawa K, Kamikubo H, Mulder FAA (2017). Unambiguous determination of protein arginine ionization states in solution by NMR spectroscopy. Angew Chemie Int Ed.

[CR50] Yuwen T, Vallurupalli P, Kay LE (2016). Enhancing the sensitivity of CPMG relaxation dispersion to conformational exchange processes by multiple-quantum spectroscopy. Angew Chemie Int Ed.

[CR51] Zeymer C, Werbeck ND, Zimmermann S, Reinstein J, Hansen DF (2016). Characterizing active site conformational heterogeneity along the trajectory of an enzymatic phosphoryl transfer reaction. Angew Chemie Int Ed.

[CR52] Zhou Z, Kümmerle R, Qiu X, Redwine D, Cong R, Taha A, Baugh D, Winniford B (2007). A new decoupling method for accurate quantification of polyethylene copolymer composition and triad sequence distribution with 13C NMR. J Magn Reson.

[CR53] Zhuravleva A, Orekhov VY (2008). Divided evolution: a scheme for suppression of line broadening induced by conformational exchange. J Am Chem Soc.

