# Supplementary material for: A 13C-detected 15N double-quantum NMR experiment to probe arginine side-chain guanidinium 15Nη chemical shifts
Source: J Biomol NMR. 2017 Nov 10;69(3):123–32. doi: 10.1007/s10858-017-0137-2 (PMC5711973; doi:10.1007/s10858-017-0137-2)
Supplement: Supplementary file 1 — Supplementary material 1 (PDF 445 KB) [file 10858_2017_137_MOESM1_ESM.pdf]

1.1 Pulse Sequence for $^{13}C^{\zeta}$-$^{15}N^{\eta}$ HDQC (Bruker)

```
; Filename: arg_cn_hdqc_hd.hm
;
; Written on 31st May 2017 by Harold Mackenzie
;
; This pulse sequence allows the recording of arginine 13Czeta-15Neta
; correlation spectra on uniformly-labelled samples. The indirect chemical
; shift reflects the double-quantum frequency of the two 15Neta nuclei.
;
; WARNING: This sequence does not work with 'getprosol'. If you by accident
; type 'getprosol' using this sequence, then all parameters must be checked
; carefully.
;
; How to:
; =======
;
; INEPT elements with 13Czeta-selective Eburp2 excitation and 15Neta-
; selective Reburp refocusing pulses:
;
;     1. Set 13C carrier (o1p) to 156.0 ppm with a 1.5 ms Eburp2
;        excitation pulse at 16.4T
;     2. Set 15N carrier (o3p) to 71.0 ppm with a 4.5 ms Reburp refocusing
;        pulse at 16.4T
;     3. Set CNST25 (15Nepsilon) to 84.0 ppm
;     4. Set CNST2 (JNC) to 20
;
;     This will generate a density element proportional to
;     4CzNz(Neta1)Nz(Neta2) after the first INEPT
;
; 13C decoupling with high-power square pulse in the middle of the 15N
; chemical shift period
;
; 1H decoupling (WALTZ64, 65us) during indirect evolution and acquisition
;
; 15N decoupling (GARP4, 350us) during acquisition

;$CLASS=HighRes
;$DIM=2D
;$TYPE=
;$SUBTYPE=
;$COMMENT=

#include <Avance.incl>
#include <Delay.incl>
#include <Grad.incl>

;DEFINE PULSES

define pulse pwc
      "pwc=p1"                              ;13C hard pulse at pl1

define pulse pwc_sele
      "pwc_sele=p11"                        ;13C selective e-pulse at pl11

define pulse pwh
      "pwh=p2"                              ;1H hard pulse at pl2

define pulse pwn
```

```
      "pwn=p3"                          ;15N hard pulse at pl3

define pulse pwn_selr
      "pwn_selr=p31"                    ;15N selective r-pulse at pl31

;DEFINE DELAYS

define delay taua
      "taua=1s/(cnst2*4)"               ;1/4JCN

"d11= 30m"                              ;Delay for disk
"d12= 20u"                              ;Delay for power switching
"d16= 200u"                             ;Delay for gradient recovery

"in0=inf1/2"

#ifdef HALFDWELL
      "d0=in0/2-pwc-0.63662*pwn"
#else
      "d0=in0-pwc-0.63662*pwn"
#endif /*HALFDWELL*/

;DEFINE OFFSETS

"cnst21=o1/bf1"                         ;Czeta
"cnst24=o3/bf3"                         ;Neta
"cnst26=0.5*(cnst24+cnst25)"            ;15N decoupling position

;DEFINE ZERO POWER ON ALL CHANNELS

"plw10=0"
"plw30=0"

;PULSE PROGRAM BEGINS

1 ze
d12 pl29:f2                             ;power to decouple(1H)
2 d11 do:f2 do:f3                       ;decoupling off
d1                                      ;recycle delay

;purge equilibrium 15N magnetisation

50u UNBLKGRAD                           ;gradient amp on
30u fq=cnst26(bf ppm):f3                ;set 15N carrier
d12 pl3:f3                              ;power to high (15N)
(pwn ph10):f3                           ;90x
2u
p51:gp1                                 ;cleaning gradient
d16

;end purge block

d12 pl10:f1 pl30:f3                     ;power to 0(13C, 15N)
30u fq=cnst24(bf ppm):f3                ;set 15N carrier, Neta

;start INEPT

(pwc_sele:sp11 ph1):f1                  ;13C selective excitation
                                        ;start Nh selective block

"DELTA = taua-2u-p52-d16-0.5*larger(pwc*2,pwn_selr)"
```

```
DELTA
2u
p52:gp2
d16 pl1:f1                                ;power to high (13C)
( center (pwc*2 ph10):f1 (pwn_selr:sp31 ph10):f3 )
2u
p52:gp2
d16
"DELTA = taua-2u-p52-d16-0.5*larger(pwc*2,pwn_selr)-0.6366*pwc"
DELTA
                                          ;end Nh selective block
(pwc ph10):f1                             ;90x

;end INEPT

d12 pl3:f3                                ;power to high(15N)
2u
p53:gp3                                   ;cleaning gradient
d16
4u cpd2:f2 ph10                           ;1H decoupling on
(pwn ph2):f3                              ;90x

;start t1 evolution period with 1H decoupling

d0                                        ;incremented delay, t1/2
(pwc*2 ph10):f1                           ;13C decoupling pulse
d0                                        ;incremented delay, t1/2

;end t1 evolution period, start second INEPT

(pwn ph10):f3                             ;90x
4u do:f2                                  ;1H decoupling off
d12 pl30:f3                               ;power to 0(15N)
2u
p54:gp4                                   ;cleaning gradient
d16
(pwc ph10):f1                             ;90x

                                          ;start Nh selective block

"DELTA = taua-0.6366*pwc-2u-p55-d16-0.5*larger(pwc*2,pwn_selr)"
DELTA
2u
p55:gp5
d16
( center (pwc*2 ph10):f1 (pwn_selr:sp31 ph10):f3 )
2u
p55:gp5
d16
30u fq=cnst26(bf ppm):f3                  ;set 15N carrier to decouple
d12 pl39:f3                               ;power to decouple(15N)
50u BLKGRAD                               ;gradient amp off
"DELTA = taua-0.5*larger(pwc,pwn_selr)-2u-p55-d16-30u-d12-50u"
DELTA
                                          ;end Nh selective block

;end second INEPT, acquire Cy magnetisation with 15N, 1H decoupling

go=2 ph31 cpd2:f2 cpd3:f3
d11 do:f2 do:f3 mc #0 to 2
      F1PH(calph(ph2,+45), caldel(d0,+in0))
```

```
exit

;PHASE PROGRAMS

ph1=  0 2
ph2=  0 0 1 1 2 2 3 3

ph10= 0
ph11= 1
ph12= 2
ph13= 3

ph31= 0 2 2 0

;DEFINITIONS

;pl1     : f1 channel - power level for hard pulse
;pl2     : f2 channel - power level for hard pulse
;pl3     : f3 channel - power level for hard pulse
;pl29    : f2 channel - power level for CPD/BB decoupling
;pl39    : f3 channel - power level for CPD/BB decoupling
;sp11    : f1 channel - shaped pulse 90 degree (selective for Czeta)
;spnam11 : Eburp2.1000
;sp31    : f3 channel - shaped pulse 180 degree (selective for Neta)
;spnam31 : Reburp.1000
;p1      : f1 channel - 90 degree high power pulse
;p11     : f1 channel - 90 degree shaped pulse        [1.5 ms at 16.4T]
;p3      : f3 channel - 90 degree high power pulse
;p31     : f3 channel - 180 degree shaped pulse       [4.5 ms at 16.4T]
;p51     : homospoil/gradient pulse
;p52     : homospoil/gradient pulse
;p53     : homospoil/gradient pulse
;p54     : homospoil/gradient pulse
;p55     : homospoil/gradient pulse
;d0      : incremented delay
;d1      : relaxation delay
;taua    : 1/(4JCN)
;d11     : delay for disk I/O                         [30 msec]
;d12     : delay for power switching                  [20 usec]
;d16     : delay for homospoil/gradient recovery
;cnst2   : J(NC)                                      [20 Hz]
;cnst21  : Czeta chemical shift offset                [= o1p, 156 ppm]
;cnst24  : Neta chemical shift offset                 [= o3p, 71.0 ppm]
;cnst25  : Nepsilon chemical shift offset             [84 ppm]
;o1p     : Czeta chemical shift offset                [156 ppm]
;o2p     : Hepsilon/Heta chemical shift offset        [7 ppm]
;o3p     : Neta chemical shift offset                 [71 ppm]
;inf1    : 1/SW = 2 * DW
;in0     : 1/(2 * SW) = DW
;NS      : 8 * n
;DS      : 16
;td1     : number of experiments
;FnMODE  : States-TPPI
;cpd2    : decoupling according to sequence defined by cpdprg2 (WALTZ64)
;pcpd2   : f2 channel - 90 degree pulse for decoupling sequence
;cpd3    : decoupling according to sequence defined by cpdprg3 (GARP4)
;pcpd3   : f3 channel - 90 degree pulse for decoupling sequence [350 us]

;for z-only gradients:
;gpz1    : 37%
```

```
;gpz2    : 11%
;gpz3    : 23%
;gpz4    : 31%
;gpz5    : 13%

;use gradient files:
;gpnam1  : SMSQ10.100
;gpnam2  : SMSQ10.100
;gpnam3  : SMSQ10.100
;gpnam4  : SMSQ10.100
;gpnam5  : SMSQ10.100

;preprocessor-flags-start
;HALFDWELL: for initial sampling delay of half a dwell-time with
;     option -DHALFDWELL (eda: ZGOPTNS)
;preprocessor-flags-end
```

1.2 Pulse Sequence for $^{13}C^{\zeta}$-$^{15}N^{\varepsilon}$-$^{15}N^{\eta}$ experiment (Bruker)

```
; Filename: arg_cn_cznenh3d_hd.hm
;
; Written on 27st April 2017 by Harold Mackenzie
;
; This pulse sequence allows the recording of arginine 13Czeta-15Nepsilon-
; 15Neta correlation spectra to aid the assignment of 15Neta DQ resonances.
;
; The flow of magnetisation is as follows:
;
; F1[13Czeta] > F3[15Nepsilon, t1] > F3[15Neta DQ, t2] > F1[13Czeta, t3]
;
; WARNING: This sequence does not work with 'getprosol'. If you by accident
; type 'getprosol' using this sequence, then all parameters must be checked
; carefully.
;
; How to:
; =======
;
; INEPT elements with 13Czeta-selective Eburp2 excitation and 15Nepsilon-
; or 15Neta-selective Reburp refocusing pulses:
;
;     1. Set 13C carrier (o1p) to 156.0 ppm with a 1.5 ms Eburp2
;        excitation pulse at 11.4T
;     2. Set CNST22 (13Cdelta) to 40.5 ppm
;     3. Set 15N carrier (o3p) to 84.0 ppm with a 6.0 ms Reburp refocusing
;        pulse at 11.4T
;     4. Set CNST25 (15Neta) to 71.0 ppm
;     5. Set CNST2 (JNC) to 20
;
;     This will generate a density element proportional to
;     2CzNz(Nepsilon) after the first INEPT and 4CzNz(Neta1)Nz(Neta2) after
;     the second INEPT
;
; 13C decoupling with a 500us adiabatic pulse during t1 and a high-power
; square pulse during the t2 chemical shift period
;
; 1H decoupling (WALTZ64, 65us) during indirect evolution and acquisition
;
; 15N decoupling (GARP4, 350us) during acquisition

;$CLASS=HighRes
;$DIM=3D
;$TYPE=
;$SUBTYPE=
;$COMMENT=

#include <Avance.incl>
#include <Delay.incl>
#include <Grad.incl>

;DEFINE PULSES

define pulse pwc
      "pwc=p1"                            ;13C hard pulse at pl1

define pulse pwc_sele
      "pwc_sele=p11"                      ;13C selective e-pulse at pl11
```

```
define pulse pwc_chirp
      "pwc_chirp=p12"                       ;13C chirp pulse at pl12

define pulse pwh
      "pwh=p2"                              ;1H hard pulse at pl2

define pulse pwn
      "pwn=p3"                              ;15N hard pulse at pl3

define pulse pwn_selr
      "pwn_selr=p31"                        ;15N selective r-pulse at pl31

;DEFINE DELAYS

define delay taua
      "taua=1s/(cnst2*4)"                   ;1/4JCN

"d11= 30m"                                  ;Delay for disk
"d12= 20u"                                  ;Delay for power switching
"d16= 200u"                                 ;Delay for gradient recovery

"in0=inf2/2"
"in10=inf1/2"

#ifdef HALFDWELL
      "d0=in0/2-0.5*pwc_chirp-0.63662*pwn"
      "d10=in10/2-pwc-0.63662*pwn"
#else
      "d0=in0-0.5*pwc_chirp-0.63662*pwn"
      "d10=in10-pwc-0.63662*pwn"
#endif /*HALFDWELL*/

;DEFINE OFFSETS

"cnst21=o1/bf1"                             ;Czeta
"cnst23=0.5*(cnst21+cnst22)"                ;13C decoupling position
"cnst24=o3/bf3"                             ;Nepsilon
"cnst26=0.5*(cnst24+cnst25)"                ;15N decoupling position

;DEFINE ZERO POWER ON ALL CHANNELS

"plw10=0"
"plw30=0"

;PULSE PROGRAM BEGINS

1 ze
d12 pl29:f2                                 ;power to decouple(1H)
2 d11 do:f2 do:f3                           ;decoupling off
d1                                          ;recycle delay

;purge equilibrium 15N magnetisation

50u UNBLKGRAD                               ;gradient amp on
30u fq=cnst26(bf ppm):f3                    ;set 15N carrier
d12 pl3:f3                                  ;power to high (15N)
(pwn ph10):f3                               ;90x
2u
p51:gp1                                     ;cleaning gradient
d16
```

```
;end purge block

d12 pl10:f1 pl30:f3                         ;power to 0(13C, 15N)
30u fq=cnst21(bf ppm):f1                    ;set 13C carrier, Czeta
30u fq=cnst24(bf ppm):f3                    ;set 15N carrier, Nepsilon

;start INEPT

(pwc_sele:sp11 ph10):f1                     ;13C selective excitation
                                            ;start Ne selective block
"DELTA = taua-2u-p52-d16-0.5*larger(pwc*2,pwn_selr)"
DELTA
2u
p52:gp2
d16 pl1:f1                                  ;power to high (13C)
( center (pwc*2 ph10):f1 (pwn_selr:sp31 ph10):f3 )
2u
p52:gp2
d16
"DELTA = taua-2u-p52-d16-0.5*larger(pwc*2,pwn_selr)-0.6366*pwc"
DELTA
                                            ;end Ne selective block
(pwc ph11):f1                               ;90y

;end INEPT

30u fq=cnst23(bf ppm):f1                    ;set 13C carrier to decouple
d12 pl10:f1 pl3:f3                          ;power to 0(13C), high(15N)
2u
p53:gp3                                     ;cleaning gradient
d16
4u cpd2:f2 ph10                             ;1H decoupling on
(pwn ph1):f3                                ;90x

;start t1 evolution period with 1H decoupling

d0                                          ;incremented delay, t1/2
(pwc_chirp:sp12 ph10):f1                    ;13C decoupling pulse
d0                                          ;incremented delay, t1/2

;end t1 evolution period, start second INEPT

(pwn ph10):f3                               ;90x
4u do:f2                                    ;1H decoupling off
30u fq=cnst21(bf ppm):f1                    ;13C carrier returned to Czeta
30u fq=cnst26(bf ppm):f3                    ;15N carrier to Ne + Nh
d12 pl1:f1 pl3:f3                           ;power to high(13C, 15N)
2u
p54:gp4                                     ;cleaning gradient
d16
(pwc ph10):f1                               ;90x
                                            ;start non-selective block
"DELTA = taua-2u-p55-d16-larger(pwc,pwn)"
DELTA
2u
p55:gp5
d16
( center (pwc*2 ph10):f1 (pwn*2 ph10):f3 );generates 4CxNh1zNh2z
2u
p55:gp5
```

```
d16
"DELTA = taua-larger(pwc,pwn)-2u-p55-d16"
DELTA
                                          ;end non-selective block
;end second INEPT

(pwc ph11):f1                             ;90y
30u fq=cnst25(bf ppm):f3                  ;set 15N carrier to Nh
2u
p56:gp6                                   ;cleaning gradient
d16
4u cpd2:f2 ph10                           ;1H decoupling on
(pwn ph2):f3                              ;90x
;start t2 evolution period with 1H decoupling

d10                                       ;incremented delay, t2/2
(pwc*2 ph10):f1                           ;13C decoupling pulse
d10                                       ;incremented delay, t2/2

;end t2 evolution period, start third INEPT

(pwn ph10):f3                             ;90x
4u do:f2                                  ;1H decoupling off
d12 pl30:f3                               ;power to 0(15N)
2u
p57:gp7                                   ;cleaning gradient
d16
(pwc ph10):f1                             ;90x
                                          ;start Nh selective block
"DELTA = taua-0.6366*pwc-2u-p58-d16-0.5*larger(pwc*2,pwn_selr)"
DELTA
2u
p58:gp8
d16
( center (pwc*2 ph10):f1 (pwn_selr:sp31 ph10):f3 )
2u
p58:gp8
d16
30u fq=cnst26(bf ppm):f3                  ;set 15N carrier to decouple
d12 pl39:f3                               ;power to decouple(15N)
50u BLKGRAD                               ;gradient amp off
"DELTA = taua-0.5*larger(pwc,pwn_selr)-2u-p58-d16-30u-d12-50u"
DELTA
                                          ;end Nh selective block

;end third INEPT, acquire Cy magnetisation with 15N, 1H decoupling

go=2 ph31 cpd2:f2 cpd3:f3
d11 do:f2 do:f3 mc #0 to 2
      F1PH(calph(ph2,+45), caldel(d10,+in10))
      F2PH(calph(ph1,+90), caldel(d0,+in0))

exit

;PHASE PROGRAMS

ph1=  0 2
ph2=  0 0 1 1 2 2 3 3

ph10= 0
ph11= 1
```

```
ph12= 2
ph13= 3

ph31= 0 2 2 0

;DEFINITIONS

;pl1      : f1 channel - power level for hard pulse
;pl2      : f2 channel - power level for hard pulse
;pl3      : f3 channel - power level for hard pulse
;pl29     : f2 channel - power level for CPD/BB decoupling
;pl39     : f3 channel - power level for CPD/BB decoupling
;sp11     : f1 channel - shaped pulse 90 degree (selective for Czeta)
;sp12     : f1 channel - shaped pulse 180 degree (adiabatic decoupling)
;spnam11 : Eburp2.1000
;spnam12 : Crp80,0.5,20.1
;sp31     : f3 channel - shaped pulse 180 degree
;spnam31 : Reburp.1000
;p1       : f1 channel - 90 degree high power pulse
;p11      : f1 channel - 90 degree shaped pulse        [1.5 ms at 11.4T]
;p12      : f1 channel - shaped pulse 180 degree       [500 us at 11.4T]
;p3       : f3 channel - 90 degree high power pulse
;p31      : f3 channel - 180 degree shaped pulse       [6 ms at 11.4T]
;p51      : homospoil/gradient pulse
;p52      : homospoil/gradient pulse
;p53      : homospoil/gradient pulse
;p54      : homospoil/gradient pulse
;p55      : homospoil/gradient pulse
;p56      : homospoil/gradient pulse
;p57      : homospoil/gradient pulse
;p58      : homospoil/gradient pulse
;d0       : incremented delay (13C-15Ne)
;d10      : incremented delay (13C-15Nh DQ)
;d1       : relaxation delay
;taua     : 1/(4JCN)
;d11      : delay for disk I/O                         [30 msec]
;d12      : delay for power switching                  [20 usec]
;d16      : delay for homospoil/gradient recovery
;cnst2    : J(NC)                                      [20 Hz]
;cnst21   : Czeta chemical shift offset                [= o1p, 156 ppm]
;cnst22   : Cdelta chemical shift offset               [40.5 ppm]
;cnst24   : Nepsilon chemical shift offset             [= o3p, 84ppm]
;cnst25   : Neta chemical shift offset                 [71 ppm]
;o1p      : Czeta chemical shift offset                [156 ppm]
;o2p      : Hepsilon/Heta chemical shift offset        [7 ppm]
;o3p      : Nepsilon chemical shift offset             [84.0 ppm]
;inf1     : 1/SW(Nh) = 2 * DW(Nh)
;inf2     : 1/SW(Ne) = 2 * DW(Ne)
;in0      : 1/(2 * SW(Ne)) = DW(Ne)
;in10     : 1/(2 * SW(Nh)) = DW(Nh)
;NS       : 8 * n
;DS       : 16
;td1      : number of experiments (Ne)
;td2      : number of experiments (Nh)
;FnMODE   : States-TPPI
;cpd2     : decoupling according to sequence defined by cpdprg2 (WALTZ64)
;pcpd2    : f2 channel - 90 degree pulse for decoupling sequence
;cpd3     : decoupling according to sequence defined by cpdprg3 (GARP4)
;pcpd3    : f3 channel - 90 degree pulse for decoupling sequence [350 us]

;for z-only gradients:
```

```
;gpz1    : 37%
;gpz2    : 11%
;gpz3    : 23%
;gpz4    : 31%
;gpz5    : 13%
;gpz6    : 41%
;gpz7    : 47%
;gpz8    : 17%

;use gradient files:
;gpnam1  : SMSQ10.100
;gpnam2  : SMSQ10.100
;gpnam3  : SMSQ10.100
;gpnam4  : SMSQ10.100
;gpnam5  : SMSQ10.100
;gpnam6  : SMSQ10.100
;gpnam7  : SMSQ10.100
;gpnam8  : SMSQ10.100

;preprocessor-flags-start
;HALFDWELL: for initial sampling delay of half a dwell-time with
;     option -DHALFDWELL (eda: ZGOPTNS)
;preprocessor-flags-end
```

2. Supporting Figure

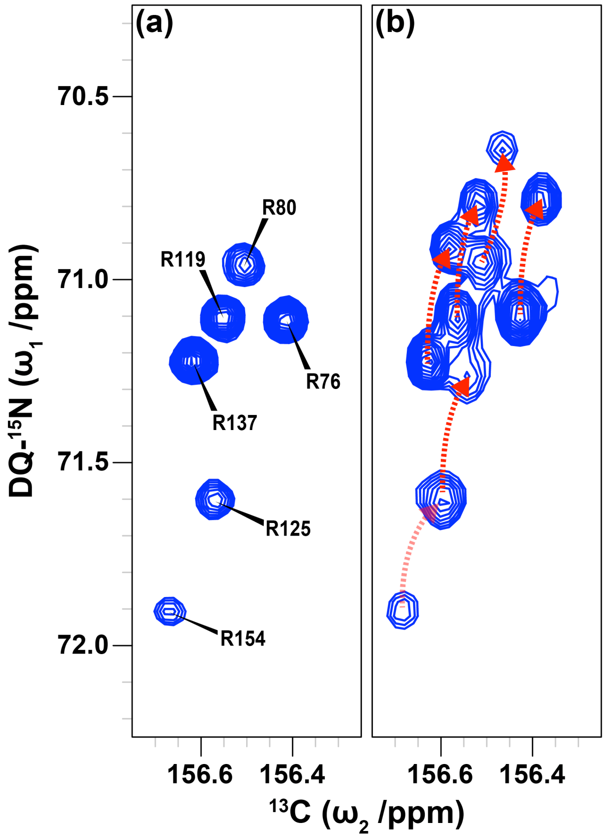

Fig. S1 $^{13}C^{\zeta}$-$^{15}N^{\eta}$ HDQC spectra of T4L99A recorded at 18.8 T at a temperature of 278K in sodium phosphate buffer at pH 5.5 containing (a) 1% or (b) 10% D_2O. At this temperature, the rotational correlation time of T4L99A (19 kDa) approaches 20 ns. The majority of the flexible arginine residues remain detectable in spectrum (a) along with the corresponding one-bond deuterium isotope shifts in spectrum (b). Analogous to R8 in Figure 5b, the singly-deuterated isotopomer of R154 overlaps with the protonated form of R125, prohibiting the characterisation of this particular shift. The spectra were acquired as (a) 512x48 and (b) 512x32 complex matrices with spectral widths of 20 ppm (^{13}C) and 10 ppm (^{15}N). 256 scans were collected for each t_1 increment with a recycle delay of (a) 2.5s or (b) 3.5s resulting in a total acquisition time for each experiment of ~17 hours.
